# A Letter from Paris

**Published:** 1875-04-15

**Authors:** C. C. Matteson

**Affiliations:** Paris


### A LETTER FROM PARIS.

EDITORS MEDICAL EXAMINER: A sufficient time having elapsed since the introduction of Koumys as a remedy in the Paris hospitals, to permit of extended observation, the profession here finds itself in a position to give a verdict upon the use of this "milk champagne" as a therapeutic agent. It has been largely experimented with by MM. Gueneau de Mussy, Gubler, Chauffard, and many others, in their hospital wards, and their researches show that koumys acts as a wonderful renovator of debilitated organisms, whatever may have been the cause of the cachexia, and that its metasyncritic action is very marked.

Though about ninety years ago, John Grieve, an English surgeon in the Russian service, called attention to the marvelous effects of koumys in all chronic diseases, the investigation was not continued, one reason being the difficulty in procuring the milk. But to-day, thanks to chemistry, we find that a mixture of asses' and cows' milk, at a certain stage of fermentation, gives about the same composition and effect.

It is in the treatment of pulmonary phthisis that a great deal of interest centres upon the koumys. The observations have been numerous in this connection, and so many cases are reported of amelioration, in every stage of the disease, that even the most skeptical have to give the subject consideration. In general the effects upon the patient may be formulated as follows: At first, a slight excitement from the alcoholic nature of the drink; this is followed by cessation of the cough, interruption of the night sweats, improvement of appetite, and marked increase in weight. In other words, general and decided improvement, and this within a few days.

To quote two cases will give an idea of the action of koumys as seen here. Dr. Labodie-Lagrave gives an observation in the *Gazette Hebdomadaire* (September, 1874), in which he states that the patient, a girl of sixteen years, was in "the last stage of pulmonary consumption." A fatal termination was expected, but under the use of koumys her strength returned, and her weight increased two kilogrammes in six weeks.

The second case that I will mention is recorded by Dr. Henri Huchard, in the *Union Medicale* for January 26th, 1875. The patient was fifty years of age, and presented the signs of pulmonary tuberculosis in the third stage (cavernous rales, etc.). For more than four months her state had been getting worse. Her weight had decreased thirty pounds. "Every day the fever was consuming her; the poor patient hardly slept, a prey to an incessant cough and to profuse sweatings; hardly any appetite, frequent vomitings, and an abundant diarrhœa had come, within a short time, to steal from the sufferer what little strength she had remaining." He ordered her one bottle of koumys (Edwards, No. 1) every day, and, the tolerance being soon established, "the sufferer saw her appetite return, her strength increase at the same time that the night sweats, diarrhœa, vomitings, and febrile exacerbations at night disappeared." In two months' time she had gained twelve and one-half pounds in weight, her face was colored, the cough was less frequent, and the lungs showed a real local amelioration. "*This patient was, without doubt, always tuberculous, but she was no longer phthisical.*"

While conversing with M. Gueneau de Mussy on the subject of koumys, he expressed to me the greatest satisfaction with its results in private practice, but regretted that the expense (three francs per bottle) prevented its constant use in the hospitals.

It is rather a discouraging task to attempt to review, in a letter, a work so full of interesting and important facts as is the one entitled, "Lectures on Anæsthetics and Asphyxia," that has lately been published by M. Claude Bernard. The name of the author is in itself a guarantee for the value of the work, and I shall only attempt to call attention to a few observations, even here finding difficulty in making a selection.

These lectures, originally delivered at the College of France during the years 1869 and 1870, have been carefully revised for publication, and have been enriched by the additional experience of M. Bernard during the past four years.

The first ten chapters are devoted to anæsthetics, not limiting the study to those substances generally named anæsthetic, as chloroform and ether, but considering the effect upon the sensitive nerves of warm water, chloral, opium and its alkaloids. The effect of the combined action of morphia and chloroform, in man as well as in the lower animals, is studied at length. It is found that—1st. If an hypodermic injection of the chlorohydrate of morphia is given an animal recovering from the effects of chloroform, the anæsthesia returns; that is, the animal becomes again insensible. 2d. On giving chloroform to an animal previously narcotized by morphia, a much less quantity is required to produce insensibility than ordinarily. Quite a number of cases are quoted where these results have been obtained in men.

In experimenting upon the tolerance of animals for opium, M. Bernard records the following curious experience, that may give a valuable indication for the general practitioner:

Having thoroughly established the tolerance in an animal so that large doses of the narcotic are borne without inconvenience, he found that it needed but a thorough *purgation* to make a new animal; that is to say, to return the animal to his first state of sensibility to the action of the drug.

Giving chloral to an animal under the influence of morphia makes the hypnotic effect more apparent, but the excitability is not diminished as by chloroform. Administering chloroform to an animal chloralized causes the sensibility to entirely disappear. Hence the conclusion that chloral, at least under these circumstances, has an action like that of morphia.

The second part of the work, comprising eight chapters, is given to the study of asphyxia, more particularly that produced by the vapors of charcoal. The author establishes, by his experiments and investigations, that the asphyxia from this cause results from the poisonment of the red blood corpuscles by the oxide of carbon rather than from the action of the carbonic acid.

To try and indicate the experimental research and carefully considered theories that the author advances on this subject would be but to translate the entire work, a task that others will doubtless perform, so I will finish this hasty notice by giving M. Bernard's outline of treatment for asphyxia, with which he closes the book:

The necessity for removal from the vitiated atmosphere and the value of artificial respiration he fully insists upon; but, in regard to the latter, he deprecates too violent efforts to establish respiration as tending to rupture the lung tissue, already more or less deteriorated following the time of exposure. The results of these ruptures would be sufficient in many cases to cause the death of the patient from secondary disease, as pneumonia, should he recover from the asphyxia. In extreme cases where cauterization may be used, the author mentions the regions over the trunks and branches of the cervico-brachial plexus as the best points of application; for these nerves, remaining longer impressionable, are able to act more directly upon the movements of respiration. The inhalation of pure oxygen and the transfusion of blood give good results practically as well as theoretically, but are generally too difficult of application.

Though but a comparatively short time has passed since this drug was introduced in Paris, Jaborandi (from the Pylocarpus Pinnatus), is now a familiar word in the wards of the hospitals. Its effects are very decidedly sudorific and sialagogue, and M. Gübler, in a recent report before the Society of Therapeutics, mentioned one case where a patient, to whom he gave 4 grammes of this medicament, sweated profusely and excreted "more than a quart (litre) of saliva." Occasionally it causes vomiting, if administered soon after eating, and when its effects are very marked, the patients complain of great thirst. Sometimes there appears tumefaction of the sub-maxillary glands and even a veritable ranula is formed.

M. Gubler sums up the principal indications for this powerful remedy as follows: Outside of anasarca or dropsy from any cause, where jaborandi gives good results, it is found that sub-acute articular rheumatism is greatly benefited by this drug. In bronchitis with asthma, it sometimes produces marvelous effects. In six cases M. Gubler arrested the access of the asthma by its use.

Following the course of new remedies, it is being used in all kinds of diseases, and its reported favorable action in diseases as widely separated as rebellious ophthalmia and polyuria requires the test of wider experience before the truth and limit of its action can be well determined. The subject is receiving a great deal of attention, and many of the medical journals are engaged in discussing the pros and cons of jaborandi. One thing seems sure, and that is that this drug has shown enough favorable action to insure its being received as a regular occupant of the Parisian medicine-chest.

The *Gazette des Hopitaux* has a custom of giving the *conclusions* that are arrived at by different writers, without burdening its pages with the details of the investigations, referring those who desire to more fully investigate the subject to the works themselves. Regarding this as a very happy custom, I shall imitate it from time to time. For example:

" Experimental Researches on the Mode of Action of the More Commonly-Employed Vomitives," by Dr. H. Chouppe (*Arch. de Phys.*). *Conclusions.* The mode of action of vomitives commonly used is not the same for all, and if we examine the phenomena that precede and follow vomiting, we will find very great differences. Ipecacuanha and its alkaloid, by whatever way they are introduced into the economy, excite vomiting by a direct irritation of the terminal points of the pneumogastric nerves in the mucous membrane of the stomach.

Tartar-emetic and apomorphine have double action; they act on the mucous membrane of the stomach, but also directly on the bulb. There is, however, a difference in these two medicaments. The former acts sooner upon the stomach than upon the bulb, while the apomorphine acts more rapidly upon the nerve centres. The best proof that can be given of this is that doses of tartar-emetic, sufficient to cause vomiting, should be greater when this substance is injected into the veins than when introduced into the stomach. With the apomorphine, on the contrary, it is by injecting into the general circulation that the maximum effect is reached.

C. C. MATTESON, M.D.

PARIS, March 10th, 1875.

